# Integrated care delivery in a rural Norwegian FACT team: an explanatory sequential mixed-methods study

**DOI:** 10.1186/s12913-026-15094-w

**Published:** 2026-07-18

**Authors:** Bjørn Steinar Storvik, Aud Uhlen Obstfelder, Anne Signe Landheim, Gry Cecilie Lunder Høiland

**Affiliations:** 1 Department of Industrial Economics and Technology Management, Faculty of Economics and Management, Norwegian University of Science and Technology (NTNU), Gjøvik, Norway; 2 Research Center for Substance Use Disorders and Mental Illness, Innlandet Hospital Trust, Brumunddal, Norway; 3 Centre for Care Research, Department of Health Sciences, Norwegian University of Science and Technology (NTNU), Gjøvik, Norway; 4 Department of Health and Nursing Sciences, Faculty of Social and Health Sciences, University of Inland Norway, Elverum, Norway

**Keywords:** Flexible Assertive Community Treatment (FACT), Community mental health services, Rural health services, Integrated delivery of health care, Mixed method research, Coordination, Interprofessional collaboration

## Abstract

**Background:**

Fragmented interfaces between municipal and specialist services make integrated mental healthcare difficult, and coordination in rural settings presents unique challenges. Flexible Assertive Community Treatment (FACT) aims to reduce fragmentation through multidisciplinary, community-based care. However, evidence remains limited regarding the services rural FACT teams deliver and how integrated care is enacted in everyday practice. This study examined service distribution and the coordination practices that enable integration in a rural Norwegian FACT team.

**Methods:**

We used an explanatory sequential mixed-methods design. A prospective service mapping captured 2,319 patient-related contacts among 70 patients over 12 weeks and was analysed with time-weighted descriptive statistics. A subsequent focus group with team members was analysed using reflexive thematic analysis. Integration occurred by building (quantitative results informed the interview guide) and merging (interpretive integration of quantitative patterns and qualitative themes).

**Results:**

Approximately 80% of services were delivered directly by the team and 20% in collaboration with external partners. Service delivery was broadly divided between clinical contacts (one-third) and interprofessional coordination/collaboration (one-third); the remaining third comprised daily life mastery, therapeutic alliance, and social support. 28% of contacts were conducted via phone, text, or videoconference, mainly for rapid coordination. Qualitative findings identified three interrelated practices underpinning integration under rural constraints: collaborative alliance, involving trust-based partnerships with patients, families, and services; information alignment, encompassing the gathering, refinement, and communication of fragmented information to establish a shared and actionable understanding; and safety valve thinking, referring to anticipating uncertainty and maintaining contingency plans to flexibly organise care within the rural network of care.

**Conclusion:**

Integrated care in rural FACT practice is enabled through extensive coordination and collaborative work, supported by relational and adaptive practices. These findings underscore the importance of context-sensitive adaptations alongside model fidelity to sustain patient safety and continuity of care.

**Supplementary Information:**

The online version contains supplementary material available at https://doi.org/10.1186/s12913-026-15094-w.

## Background

Patients living with complex problems and long-term needs require interdisciplinary treatment and coordinated cross-sectoral support, yet despite successive reforms and the introduction of new coordination models, ensuring robust and effective coordination practices across municipal and specialist levels remains a persistent challenge in everyday care delivery [1, 2]. Poor coordination is linked to reduced quality and safety of care, reinforcing repeated calls to restructure how healthcare delivery is organised to better support integrated and reliable care processes [3–5].

Providing integrated and coordinated care for patients living with severe mental illness (SMI), often with co-occurring substance use disorders (SUDs), is inherently complex, as their care trajectories involve fluctuating needs, multiple providers, and continual interface work across organisational boundaries [6, 7]. These challenges take on distinct forms in rural areas [8, 9], where limited access to specialised services, geographic dispersion, and resource constraints shape everyday care delivery to support patients with complex needs [10, 11]. In this study, rural is defined according to the national classification provided by Statistics Norway (SSB), using the centrality index, which ranks Norwegian municipalities into six levels based on access to employment and services. Municipalities classified in the lower centrality categories represent rural contexts. This classification constitutes the standard operationalisation of rurality in Norwegian health services research [12].

Rather than compartmentalizing different aspects of care across multiple service providers, as often seen in traditional service models, multidisciplinary service models have increasingly gained prominence as means of delivering integrated care to meet the diverse care needs of patients living with SMI [13]. Flexible Assertive Community Treatment (FACT), developed in the Netherlands [14] is one such model. FACT was introduced in Norway in 2013 [15], one year after a major reorganisation of the healthcare system that shifted responsibility from specialist to municipality health services including a substantial reduction in the number of inpatient psychiatric beds [16]. In Norway, the FACT model was introduced as a policy response to operationalize integrated care for patients living with SMI, particularly targeting individuals who had fallen outside or not benefited sufficiently from existing service structures [15]. The model was intended to bridge municipal and specialist mental healthcare services through coordinated, multidisciplinary team-based care. Accordingly, we draw on a definition of integrated care, where the concept of care is understood holistically [17], as “an approach that seeks to improve the quality of care for individual patients, service users and carers by ensuring that services are well coordinated around their needs” [18].

FACT [14] combines principles of individual case management with Assertive Community Treatment (ACT) [19] to provide services to all patients living with SMI, supporting recovery in areas of life that are important to them [15]. A core feature of FACT is its flexible intensity of care, which enables teams to dynamically upscale or downscale services in response to patients’ fluctuating needs. During crisis and periods of increased need, an ACT mode of care is temporarily activated, in which services are provided by the entire team and discussed daily in whiteboard meetings [20]. In more stable phases, a designated case manager (CM) retains primary responsibility for individual care, while selectively involving other team members as needed [15, 21]. In Norway, implementation and auditing of FACT are still based on the 2010 fidelity scale [22], rather than the later 2017 R version [23]. Importantly, the FACT model itself has remained largely stable over time, while the 2017 version was intentionally developed to better support teams operating in an interagency context. Differences between the Norwegian 2010 fidelity framework and the later 2017 R-FACT scale should therefore be understood as reflecting differences in measurement and implementation context, rather than changes in the underlying model. Within the Norwegian fidelity framework, outreach constitutes a core quality indicator, with high fidelity associated with cases in which approximately 80% or more of face-to-face contacts occur outside the team office [22].

FACT teams in Norway are mostly organized as a binding collaboration between the municipality and specialist mental health services and operate under two legal frameworks [15]; specialist care is state-administered, while municipalities are responsible for primary mental health services [24]. Norwegian FACT teams are supposed to provide the bulk of services from the team [15]. However, some services fall outside the mandate of Norwegian FACT teams, such as primary care provided by general practitioners and parts of somatic specialist care [15]. Consequently, Norwegian teams must combine service delivery from the team while ensuring access to and coordinating services they are unable to deliver in collaboration with external services. Although some national and local adaptations to the FACT model have been made, the inherent complexity and fragmentation of the Norwegian healthcare context limit the extent to which potensial of core FACT principles can be utilized [8, 25].

Facilitators of successful integrated care in rural areas include locally co-designed models, clear role definitions, co-location, and strong leadership [26, 27]. However, rural barriers, such as workforce instability, funding insecurity, fragmented governance, professional silos, rural place stigma, and technological limitations, continue to undermine efforts to create cohesive and responsive systems of care [28, 29]. These challenges are further reinforced by governance structures that do not fully account for rural conditions, complicating efforts to align services across sectors [30].

Norwegian rural FACT teams also face persistent structural barriers including fragmented service systems, cross-level legal constraints, and collaboration challenges [8, 31]. Empirical studies indicate that coordinating external services is particularly demanding for these teams, as integration work must be performed across organisational boundaries and administrative levels [31]. A key technical constraint relates to uneven implementation and limited interoperability of electronic health record systems across municipal and specialist services, which impedes information sharing and day-to-day coordination [32].

Empirical studies of Norwegian rural FACT teams describe adaption to these constraints through context-adaptive planning, large geographic catchment areas, redused caseloads, delegation of tasks to municipal services, use of digital tools, and flexible staff location [8, 25]. Notably, one study suggests that coordination work is particularly time-consuming in Norwegian rural FACT teams, reflecting the complexity and fragmentation of the surrounding service system [8]. Additional measures include multidisciplinary meetings, family involvement, and the systematic use of standardized tools to support integrated practice and alignment with the FACT model [31, 33]. Collectively, these findings suggest that rural FACT teams rely on both intrateam adjustments and cross-sector coordination to keep care trajectories coherent despite system-level constraints.

Despite the growing use of FACT teams in rural mental health services, little is known about how team members within these teams actually work, what services they deliver, and how care is integrated within teams and through coordination with external services [34]. To our knowledge, there are only two studies that have examined the actual contacts and work of employees in a FACT team [35, 36]. However, little is known about the actual distribution of services and everyday coordination practices of rural FACT teams. This knowledge gap is particularly relevant as policy and fidelity frameworks assume integrated delivery, whereas rural Norwegian teams operate under dual legal regimes and constrained digital and organisational infrastructures. Robust evidence on actual service patterns and coordination work is needed [37], and should account for how the rural contexts [11] and relational factors [38] influence team dynamics.

Norwegian rural FACT teams provide a particularly relevant context for studying integrated care. Norway’s cross‑level service structure, combined with rural conditions such as long distances, low population density, and limited access to specialist services, render the coordination demands the FACT model is designed to address. Against this backdrop, this study examines both *what* services a rural FACT team provides, as reflected in patient-centred contacts and patient-time, and *how* integrated care is made possible in everyday practice. The aim of this study is to examine how a rural Norwegian FACT team delivers and integrates services in everyday practice by (1) identifying the distribution and nature of patient-centred services provided by the team and in collaboration with external partners, and (2) explaining how team members’ coordination and collaboration practices enable integrated care within the constraints of a rural context.

To address this aim, the study adopts an explanatory sequential mixed‑methods design. This design allows us first to examine the range and distribution of services provided by the team including those delivered in collaboration with external partners; second, we explore how these services are operationalized and integrated into everyday practice. This design therefore makes it possible to understand not only the scope of patient-centered service provision, but also how the team’s collaborative and coordination practices help explain the service patterns observed in the quantitative phase.

## Methods

### Study design

This study adopted a sequential explanatory mixed-methods design [39], combining quantitative and qualitative approaches to investigate integrated service provision within a rural Norwegian FACT team. The quantitative phase mapped the distribution and nature of services delivered by the team, both independently and in collaboration with external providers. The qualitative phase then explored how team members’ coordination and collaboration practices, informed by patterns identified in the quantitative analysis, were operationalized in everyday work to enable integrated care in a rural context. Informant sampling was consistent across phases.

This design, as advocated by Creswell and Creswell [39] and by Demkowicz et al. [40], was particularly suited to illuminate the scope, content, and modalities of service provision, and how these are organized to enable coherent services for patients living with SMI.

Mixed-methods integration followed the framework described by Fetters et al. [41] and occurred at two points in the study. First, building was achieved as findings from the quantitative phase directly informed the development of the interview guide for the focus group. The guide was developed specifically for this study and is provided as a supplementary file (Supplementary file 1). Second, merging took place during the interpretation of results, where quantitative patterns and qualitative themes were brought together to understand how service provision and coordination practices enabled integrated care in a rural setting.

### Setting and characteristics of the participating FACT team

This study consists of one FACT team in a rural coastal region in Norway consisting of multiple small municipalities, with a sparsely populated area spread across islands and an extensive geographic catchment. The team operates under a formal collaboration agreement between four municipal mental health services and a District Psychiatric Centre (DPS), and is organised across two local offices to manage geographical dispersion. The team covers around 19,000 inhabitants aged 18 and older in the catchment area. The four municipalities served by this rural FACT team range from around 1,000 inhabitants in the smallest to approximately 10,000 in the larger municipalities.

Geographically, the municipalities are aligned linearly across the islands, with up to 3.5 h of driving from one end of the catchment area to the other under normal conditions. The nearest psychiatric inpatient unit is located approximately 6–7 h of travel away, and acute psychiatric admissions therefore require transport by air ambulance. This spatial dispersion, combined with ferry connections, limited public transport, and weather-dependent road and aviation conditions, creates structural barriers to service accessibility and continuity of care. During the study period, the team consisted of 14 staff members with a lower effective full-time equivalent (FTE) due to shared positions, corresponding to 9 FTEs. Staff were employed across municipal and specialist levels, with a mixed employer composition. The team included psychiatric nurses, nurses, social workers, a psychologist, a psychiatrist, an IPS employment specialist, a peer specialist, and personnel with substance-use expertise; a formal rehabilitation specialist role was not established.

The team maintained a low caseload, increasing to 70 users by 2024. The daily whiteboard meetings and the weekly treatment meetings were conducted through a combination of in-person attendance and videoconferencing between the two offices. Each patient had an individualized treatment plan and a dedicated CM, in line with the national handbook [15].

The participating FACT team achieved high overall fidelity, with a total score of 4.3 (2022), indicating *very good model fidelity*. Six of seven main fidelity domains were rated *very good* at the assessment.

### Participants

The study employed purposive sampling to select an established, high-fidelity FACT team operating in a rural context. All 14 team members were invited to participate in both phases of the study. While all team members participated in the service mapping, nine of the same informants, who were available on the scheduled interview date, participated in the focus group interview.

### Data collection and measurements

#### Quantitative data collection

The quantitative data consist of services delivered by the FACT team independently and in collaboration with external services. These data were collected through a structured service-mapping procedure in which all team members prospectively registered each patient-centered contact during the 12-week data-collection period in 2024. At the time of data collection, the team served 70 patients. Contacts were defined as episodes of professional patient-centered activity, with or without patient presence. Routine internal meetings (e.g., whiteboard meetings) were not recorded. The mapping encompasses all contacts conducted by one or more team members with or about patients. The mapping does not show the total working time of the team members.

Each contact was documented using a structured registration sheet developed for the study (Supplementary file 2). For each entry (contact ID), the following attributes were recorded: date, location (including technology-mediated contacts), patient ID, content (main and up to two additional categories), participants (including other team members and external providers), duration, and travel time (if applicable). To ensure consistency, location, content, and external participation were coded using predefined categories.

#### Qualitative data collection

The qualitative data were collected through a focus group interview [42]. While the service mapping provided descriptive insights into the distribution and resource allocation of service provision, it could not capture the underlying patterns or contextual rural factors shaping interprofessional collaboration and coordination that made integration of services possible. Patterns identified in the service mapping were presented to the informants in advance of the focus group interview. The results of the quantitative analysis, especially unexpected findings, guided the development of the focus group interview guide [42]. The focus group interview took place one year later and was conducted at one of the offices using a hybrid format, with team members at the other office participating via videoconference. No staff turnover occurred during the intervening period. The focus group interview lasted 1.5 h, was audio-recorded, and later transcribed. The purpose of the focus group was to explain key quantitative patterns in service delivery and collaboration by eliciting team narratives about how coordination and cross-service integration are enacted in everyday work in the rural FACT setting; the interview guide was derived from the quantitative results and cross-checked against the FACT model manual and fidelity scale [15, 22].

### Data analysis

#### Quantitative data analysis

Descriptive data analysis was conducted using IBM SPSS Statistics 23. Over a 12-week period, 14 members of the rural FACT team recorded a total of 2,427 patient-related contacts involving 70 patients. Cases with missing values for location, content, or duration (*n* = 108) were excluded, resulting in a final dataset of 2,319 contacts. Total time spent on all contacts was 1072 h (64,297 min). The main content codes were reorganized into seven thematic content categories. All percentages are calculated relative to total recorded patient-centered episode time and do not represent proportions of staff-working hours or workforce capacity.

##### Estimation of face-to-face contacts and outreach

Outreach was estimated in accordance with the FACT Fidelity Scale 2010 (item 50) [22] as the proportion of face-to-face contacts occurring outside the team-affiliated office. Face-to-face contacts (in Table 1) were defined as *all community-based contacs*, and the thematical contents *social and leisure*, *daily life mastery*, *clinical and treatment*, *did not attend/no* contact that took place at *the teams affiated office* and at *external service provider offices.*

##### Travel time

Travel time was analysed and reported separately from patient centred time, reflecting the predefined distinction between patient-centred and non patient-centred activities.

##### Internal service provision

Relative time spent per content category and location was calculated using the MEANS procedure with WEIGHT BY time-use, and cross tabulated. Resource allocation is presented in Table 1, showing the distribution of time use (%) by location and content category. This approach was chosen to capture the intensity of patient-centered services, as time use provides a more accurate measure of resource distribution than contact frequency.

##### Services provided in collaboration with external services

Relative time spent in collaborative contacts per content category and collaboration partners was calculated by weighing the variable representing time spent. We performed a cross tabulation, with row percentages displayed, and the same procedure was repeated with column percentages. Table 2 shows how time is distributed across different service content categories and external collaboration partners.

#### Qualitative data analysis

Data from the focus group interview were analyzed based on a reflexive thematical analysis [43] to identify patterns in the complex, interactional coordination and collaboration practices of a rural FACT team. The process began with familiarization, involving thorough reading of the transcript. NVIVO software [44] was used to organize data, which improved the transparency and structure of the analysis. The analysis followed an inductive approach, first focusing on interactional patterns across different FACT episodes and collaborative arenas. Through iterative coding and comparison, we identified a set of crucial organising elements, which were subsequently synthesised into three key practices for coordination and collaboration: *collaborative alliance*,* information alignment* and *safety valve thinking*. Together, these practices describe how team members mobilized care trajectories tailored to the rural context.

## Results

In the following, we first present the results of the service mapping assessing the distribution and resource allocation of patient-centered services provided by the rural FACT team, followed by patient-centered services provided in collaboration with external service providers. We then present the qualitative findings, which explore how team members coordinate and integrate care in their day‑to‑day work.

### Quantitative findings: distribution of services and collaboration patterns

#### Services provided by the FACT team

Table 1 presents the distribution of relative service time by location and service content for 2,319 registered patient-related contacts in the rural FACT team. Total time spent on all contacts was 1072 h (64,297 min), of which 286 h (398 contacts; 17.2%) involved two team members and 68 h (86 contacts; 3.7%) involved three.

**Table 1:** Time-weighted distribution of patient-centered service time (% of total recorded patient time) by location and service content, with contact counts (*n* = 2,319)

Location	Total time per location (% of total patient-centered service time) (Total number of contacts per location)	Therapeutic alliance with patient and network	Social and leisure activities	Daily life mastery	Clinical contacts and treatment	Interprofessional coordination and collaboration	Did not attend/no contact	Other
Community-based contact	37.6% (438)	3.4% (58)	5.6% (60)	6.7% (82)	18.8% (180)	2.0% (32)	0.9% (21)	0.2% (5)
Team-affiliated office	31.8% (469)	1.8% (20)	0.7% (5)	1.4% (16)	7.3% (89)	18.4% (304)	0.6% (12)	1.6% (23)
External service provider office	2.6% (27)	0.2% (3)	0.0% (0)	0.7% (9)	0.8% (5)	0.4% (8)	0.0% (0)	0.5% (2)
Phone call	19.7% (873)	0.8% (48)	0.3% (16)	1.4% (85)	5.5% (200)	10.6% (460)	0.3% (27)	0.8% (37)
Text message	5.0% (471)	0.5% (43)	0.1% (12)	0.3% (30)	0.9% (56)	2.9% (309)	0.2% (9)	0.1% (12)
Video-conference	3.3% (41)	0.1% (1)	0.0% (0)	0.0% (0)	0.3% (3)	2.8% (36)	0.0% (0)	0.1% (1)
Sum contents (%)	100% (2319)	6.8% (173)	6.7% (93)	10.5% (222)	33.6% (533)	37.1% (1149)	2.0% (69)	3.3% (80)

Note. Percentages are calculated as proportions of total patient-centered service time (1,072 h; 64,297 min). Data are based on 2,319 registered contacts. Percentages may not sum to 100.0% due to rounding. Contact counts per cell are shown in parentheses.

##### Overall time by location

The largest share of time was spent in community-based settings (37.6%; average duration 55 min), and at team-affiliated offices (31.8%; average duration 44 min), followed by phone contacts (19.7%; average duration 15 min). Text messages (5.0%; average duration 7 min), videoconferences (3.3%; average duration 52 min), and contacts at external service providers (2.6%; average duration 60 min) accounted for smaller proportions. Taken together, phone contacts, text messages, and videoconferences accounted for approximately 28% of total patient-centered service time.

##### Overall time by service content

Across locations, interprofessional coordination and collaboration represented the highest share of time (37.1%; average duration 21 min), followed by clinical contacts and treatment (33.6%; average duration 40 min) and daily life mastery (10.5%; average duration 30 min). Therapeutic alliance activities accounted for 6.8% (average duration 25 min), while social and leisure activities represented 6.7% (average duration 46 min). Non-attendance/no contact and other activities accounted for 2.0% (average duration 18 min) and 3.3% (average duration 27 min), respectively. Within the category of clinical contacts and treatment, family sessions (code 12) constituted 3.2% of total time, with an average duration of 40 min.

While about half of the coordination and collaboration was conducted in the office, the remainder of this work was carried out via phone calls, text messages, and videoconferences.

##### Estimation of face-to-face contacs and outreach

Based on the predefined criteria, face-to-face contacts comprised an estimated 574 encounters (25% of all contacts), but accounted for 49% of the total patient-centred time. Outreach was estimated at 78.7%.

##### Travel time

The total recorded travel time amounted to 252 h, based on 449 contacts with registered travel time. 19.4% of contacts involved travel, and among these, most travel durations were short, with the majority lasting 20 min or less. No travel time was included in the majority of contacts (80.6%).

#### Service provision in collaboration with external services

Among all registered contacts, 19% (206 h; *n* = 417) involved collaboration with external partners. The time-weighted distribution of collaborative patient-centered service delivery (19% of total time) is presented in Table 2.

**Table 2:** Time-weighted distribution of patient-centered service time (19% of total time) delivered by the FACT team in collaboration with external services, by partner and content category

Collaboration partner	Total % of collaborative time	Therapeutic alliance with patient and network	Social and leisure activities	Daily life mastery	Clinical contacts and treatment	Interprofessional coordination and collaboration	Did not attend/ no contact	Other
Other services	24.2%	0	0	1.6%	2.3%	1.3%	0	4.0%
GP	17.3%	0.5%	0	2.6%	5.8%	8.4%	0.1%	0
Family and next of kin	15.1%	0.4%	0	0.8%	5.9%	7.2%	0.5%	0.3%
NAV (Norwegian Labour and Welfare Administration)	9.6%	0.2%	0	4.6%	0.2%	4.2%	0.2%	0.1%
Inpatient unit at DPS or hospital	8.6%	0.5%	0	0	0.3%	7.3%	0.5%	0
Municipal home care services	6.3%	0	0	0.3%	0.8%	5.1%	0.1%	0
Municipal mental health and substance use services	5.5%	0.6%	0	0	0.8%	4.0%	0	0
Outpatient and acute services at District Psychiatric Centre (DPS)	3.2%	0	0.2%	0	0.5%	2.5%	0	0
Emergency primary care services	3.1%	0	0	0	2.6%	0.5%	0	0
Police and correctional service authorities	2.1%	0	0	0	0.8%	0.5%	0	0.7%
Employers	2.0%	0	0	1.9%	0	0.1%	0	0
Somatic hospital	1.5%	0	0	1.5%	0	0	0	0
Dentist	0.9%	0	0	0.5%	0.3%	0.1%	0	0
Substance use outpatient clinic, including opioid agonist treatment (OAT)	0.4%	0	0	0	0	0.4%	0	0
Psychiatrist / psychologist in private practice	0.3%	0	0	0	0	0.3%	0	0
Total	100.0%	2.2%	0.2%	13.9%	20.3%	56.9%	1.3%	5.1%

Note. Percentages are calculated on collaborative patient-centered service time (206 h), based on 417 collaborative contacts; rows/columns may not sum to 100.0% due to rounding. Categories reflect time-weighted distributions by collaboration partner and service content

##### Overall time by service content (collaborative contacts)

Interprofessional coordination and collaboration accounted for the majority of time use, representing 56.9% of the total. Clinical contacts and treatment constituted 20.3%, while daily life mastery represented 13.9%; the remaining content categories contributed relatively small shares.

##### Overall time by collaboration partner

The largest proportion of collaborative time was spent with other services (24.2%; e.g., emergency medical communication centre, legal advisor, supervisory commission, child welfare services, inpatient SUD treatment facility), followed by GPs (17.3%) and family and next of kin (15.1%). Collaboration with NAV (Norwegian Labour and Welfare Administration) accounted for 9.6%, and inpatient unit at District Psychiatric Centre (DPS) or hospital represented 8.6%. Smaller shares were contributed by municipal home-care services (6.3%), municipal mental health and substance use services (5.5%), outpatient and acute services at DPS (3.2%), and emergency primary care services (3.1%); other partners contributed marginally.

##### Cross-tab highlights (partner × content)

Overall, interprofessional coordination and collaboration predominated across nearly all external partners (56.9%). Notable exceptions included emergency primary care services, where collaborative contacts were chiefly clinical (2.6%), and NAV and employers, where daily life mastery dominated (4.6% and 1.9%, respectively).

With GPs, 5.8% of collaborative time involved clinical work, in addition to coordination and collaboration (8.4%). Family members were involved in both clinical and collaborative activities. Family sessions accounted for 3.2% of the total recorded time and were included under clinical contacts and treatment (Table 1). In addition, contacts involving family members constituted 15.1% of the time categorized as coordination and collaborative work (Table 2). Taken together, interactions involving family members accounted for 5.4% of the total recorded time (adjusted for duplicates).

### Bridging quantitative with qualitative findings: from service patterns to practices

Across services provided by the team (Table 1) and those delivered in collaboration with external partners (Table 2), we observed that the team’s patient-centered time use was broadly divided between clinical contacts and interprofessional coordination and collaboration, each accounting for approximately one-third of total time. The remaining third comprised activities related to daily life mastery, therapeutic alliance, and social support. Services delivered in collaboration with external partners represented approximately 20% of total time and were largely dominated by coordination and collaboration tasks, particularly with municipal services, GPs, and relatives.

An overarching pattern in the service-mapping data is that a substantial share of patient-centred time was devoted to interprofessional coordination and collaboration (37%), primarily conducted in the office, and extended through extensive use of digital communication (28%). The majority of face-to-face contacts took place outside the office, with an estimated outreach rate of 79%. Nevertheless, travel was included in only 20% of contacts, which were predominantly brief, lasting less than 20 min. This distribution raises questions about how such integrative work is carried out in practice. While the quantitative findings show what services the team provides and where it takes place, they do not explain how or why these patterns emerge. The qualitative analysis therefore explores the routines and practices that enable organization and integration of care in a rural context.

### Qualitative findings: routine practices that enable integrated care

Drawing on the interview data, we identified three routine practices that illuminate the work behind the mapped service patterns and explain how FACT team members collaborate and coordinate care for patients living with SMI: *collaborative alliance*, *information alignment* and *safety valve thinking*.

#### Collaborative alliance: building and maintaining the relational infrastructure of care

Collaborative alliance refers to the relational and structural work through which the FACT team establishes and sustains trust‑based working relationships with patients, relatives, and external partners. It captures how team members described cultivating accessibility, familiarity, and mutual responsiveness as the relational foundation for coordinated action.

The team members highlighted that the FACT model required more than procedural compliance, as several team members noted, it demanded a fundamental shift in practice, from individual to team-based care. Team members acknowledged that this transition was challenging, and, according to them, ingrained habits of working independently and assumptions that team involvement was optional persisted. Staff explained, especially during early implementation of the FACT model, that aligning multiple professionals around a single patient required deliberate effort, follow-ups and conscious investment in collaborative routines to normalize joint responsibility. There was consensus, the team members explained, that the CM held the overarching responsibility for orchestrating mediation and advocacy work to build and sustain these alliances, thereby anchoring trust and mutual respect.

The team members found it foundational to obtain a comprehensive overview of potential external collaboration partners involved in each patient’s care. Despite the time investment, they regarded this work as essential for establishing respectful relationships with patients and their families, as well as for building effective alliances with colleagues and external service providers. All members, including relatives, of the alliance were incorporated into the patient’s individual treatment plan. One team member emphasized that the strength of the working relationship within the alliance shaped how smoothly collaboration unfolded in practice, helping those involved in the patient’s care to develop a collective understanding of the patient’s situation:

I have experienced a difference between trajectories “where you come straight off the ski jump”, where the treatment plan is there as a well-prepared document, in contrast to the trajectories where you “start off on the wrong foot”. There is a significant difference between these two types of trajectories.

Team members explained that the phone was frequently used to establish a relationship, and they also valued text messages for building trust and establishing a therapeutic relationship, especially with patients in vulnerable phases. They further reported that the peer specialist was used as a valuable resource for initial contact when team members perceived the establishment of a therapeutic relationship as challenging.

While collaborative alliance provides the relational infrastructure for joint work, we found that integrated care also depends on how information is collected, translated and aligned across actors.

#### Information alignment: making heterogeneous information coherent

Information alignment refers to the collaborative informational work through which team members gather, refine, and communicate fragmented information so that involved actors can work from a shared and actionable understanding of the patient’s situation. While collaborative alliance provides the relational foundation for joint work, information alignment captures the cognitive and organisational efforts required to maintain coherence across assessments, priorities, and responsibilities in everyday care.

Team members expressed that the collection and flow of information, especially from external providers, was challenging and resource intensive due to the incompatibility between digital systems. The informants claimed that to obtain and maintain an overview, information had to be collected manually, often through phone calls to various external partners.

As one informant explained, the process typically entailed three steps: “I collect the information, I often re-shape it, and then re-tell it [to team members and collaboration partners]”. The staff agreed that the CM held the primary responsibility for maintaining a comprehensive overview of flow of information, including sharing relevant information with other team members. According to the informants, this also meant aligning different perspectives, diagnoses, risks, priorities, and planned next steps, ensuring that decisions and responsibilities remained coherent across services. Informants emphasized the unique contribution of the peer specialist, whose input from a patient perspective enriched the team’s understanding. When information was aligned and responsibilities clarified, the informants continued, they documented this overall aim in the treatment plan. They considered the treatment plan, with an overview of involved actors in the alliance, as a common frame for further action.

The team members consistently emphasized the importance of involving the patient and their family in the formulation of the treatment plan, although this aspiration was not always realized in practice. Nevertheless, staff expressed that the treatment plan functioned as a strategic framework, continuously guiding and modulating care efforts toward the attainment of the goals articulated within the plan.

I have experienced that it’s easier [to organize care] when there is a solid treatment plan in place. However, I haven’t managed to achieve that equally well for all my patients.

Team members explained that communication practices were shaped by both technological affordances and relational dynamics, grounded in the strength or weakness of ties within the collaborative alliance, as well as by additional situational factors such as urgency of the situation and available tools. When they were out of the office, as one informant put it, “on the road, the phone is the only thing you have”. Team members explained that they valued text messages for their low-threshold nature, and for their practicality in “quick co-ordinational tasks”.

Several informants described these practices as diverse and pragmatic, taking place through a combination of formal structures, such as whiteboard meetings, and informal interactions, including hallway conversations and electronic messages. Furthermore, the team used a supplementary “back board,” as referred to by the informants, on which all patients were listed and reviewed during team discussions at the end of each whiteboard meeting when considered necessary. They also reported that patients living in the two most peripheral municipalities, referred to by the team as “FACT satellites”, were always listed on the whiteboard. According to the informants, this arrangement reflected the importance of maintaining shared situational awareness of patients, as articulated by one of the team members: *“Without the board*,* I would only have knowledge of a limited number of patients. The board brings the team together around a table and provides insight into other team members’ work with patients”.*

Mobile phones and text messaging were described as supplements to in-person contacts and enabling more to the point communication, and staff found it as a good investment, because thorough planning and achieving and maintaining an overview of the flow of information allowed them to spend their time more efficiently: “*we would never be able to do our actual work without the phone!”* Typical scenarios included making a call or sending SMS reminders to patients about an appointment later the same day, or establishing mutual agreements that the patient could call, should the need arise, as an optional add-on between consultations. Team members further reported that SMS was the preferred mode of communication with relatives. Team members noted that collaboration partners often refrained from contacting team members directly and instead chose to route their calls through the main switchboard, and they expressed uncertainty about the reasons behind this practice.

Even when information is aligned and responsibilities clarified, the unpredictable nature of patients’ needs and rural service availability requires the team to work proactively to manage uncertainty.

#### Safety valve thinking: proactive modulation of care under uncertainty

*Safety valve thinking* reflects the team’s proactive approach to managing uncertainty and mitigating risk in patient care, and involves coordination as well as collaborative work. The informants described a mindset embedded in their daily routines, where contingency planning and flexible responses were prioritized to safeguard both patients and staff. Safety valve thinking involves the capacity for dynamic adjustment in response to changing care needs.

All the informants explained that the overarching objective of FACT team staff members day-to-day work was to achieve the overall goals outlined in each patient’s individualized treatment plan whilst maintaining coherence across the various components of care. However, as one of the informants in the focus group interview noted: *“The path to achieving these goals – in practice – is not clearly outlined in the treatment plan”.* Team members described that, in order to manage the realities of everyday care, flexibility and improvisation were often required. This was underscored by one informant, who stated: *“I never regret not following the fidelity scale – the patients are more important than the model”.* This comment referred to the fact that the team operated only during weekday hours (08:00–16:00). Another team member illustrated this by posing a rhetorical question: *“What do we then do if the plane can’t take off because the weather won’t allow it*,* and the patient needs urgent hospital admission?”*

The team members emphasized a unanimous consensus regarding the role and responsibilities of the CM. According to them, this role was characterized by flexibility and adaptability, and included ensuring continuity of care, coordinating multidisciplinary interventions, monitoring progress toward treatment goals, and facilitating communication between the patient, relatives, team members, and external service providers. As expressed by one of the informants: this involved close monitoring, functioning as a “safety valve”. Another noted that in some situations, the CM would take a more distant position while still safeguarding continuity. A third informant captured the role metaphorically: *“It’s like being a chef! You gather the ingredients*,* organize the staff in the kitchen*,* and in the end it’s you*,* as the chef*,* who puts it all together in the pot.”*

In more stable trajectories, the informants explained that the CM held the overarching responsibilithy for maintaining oversight. However, other team members and external partners were involved when needed. Staff members described valuing the mobilization of the team’s multidisciplinary resources and facilitating joint decision-making through what they referred to as emergent “mini-meetings.” These meetings were described as being organized based on situational and contextual assessments of what informants referred to as a “best match,” meaning that colleagues were strategically selected according to the specific expertise required to address the issue at hand.

The team members reported that more complex trajectories were discussed collectively in the team, facilitating formulation of hypotheses, exchange of perspectives, and the design of tailored interventions. The informants valued whiteboard meetings, and they expressed that these meetings were a central arena for interdisciplinary reflection and oversight. After reaching a rough consensus on the patient’s situation, they explained that they would coordinate who would do what, where, and when. According to the informants, if this required minimal time, agreements were finalized during the whiteboard meeting, otherwise, coordination of planned activities would continue after the whiteboard meeting. Additionally, these meetings functioned as a “fail-safe” solution: as one informant noted, colleagues would occasionally remind him if tasks were completed, ensuring accuracy and continuity.

The team members explained that the degree of involvement of the CM varied depending on the patient’s needs, the complexity of the case and the situation at hand, and also underscored that the approach was continuously and dynamically adjusted. The informants explained that they were also tasked with practical responsibilities such as organizing appointments, supporting patients in administrative processes (e.g., applications for housing or financial assistance), and ensuring adherence to medication and therapeutic regimens. The informants also mentioned a locally embedded practice within the team: when the CM was unavailable, colleagues could temporarily assume the role, ensuring that the primary CM was promptly updated upon their return. This form of collegial substitution was enabled by strong interpersonal familiarity and a foundation of trust within the team.

One of the informants mentioned an example of the inefficiency they sometimes faced, where assisting a patient in need of staffed housing, resulted in a cycle of meetings without substantive progress, forcing the entire team to invest significant time and effort merely to secure the patient’s basic services. The informant explained that despite repeated applications and recommendations from inpatient units and the FACT team, and according to him, municipal authorities had delayed decisions, citing financial limitations. Ultimately the municipality resorted to purchasing a private solution, but only after prolonged negotiation. Another informant described this dynamic as leaving the team “holding the short end of the stick”, bearing responsibility for patient safety and continuity while lacking the authority to implement necessary measures.

Together, these three practices explain how the FACT team makes integrated care operational: collaborative alliance provides the relational foundation, information alignment creates a shared cognitive frame, and safety valve thinking enables flexible, situation specific adjustment of care arrangements.

## Discussion

This study examined what services a rural Norwegian FACT team provides and how team members work to enable integration of care in everyday practice.

Our analysis of service provision showed that this rural Norwegian FACT team delivered a comprehensive package of care and support, with approximately 80% of services provided directly by the team and around 20% delivered in collaboration with external partners. Services delivered by the team were distributed almost evenly across clinical contacts (one-third) and interprofessional coordination and collaboration (one-third), with the remaining third devoted to daily life mastery, therapeutic alliance, and social or community-oriented activities.

Patient-centred service delivery accounted for 1,072 h during the study period. Of this time, 286 h involved two team members and 68 h three. A further 252 h were spent on travel. The remaining time was primarily devoted to team-based activities not captured in the contact dataset, including FACT board meetings, treatment meetings, administrative work, breaks, and other core functions inherent to routine service delivery.

The difference between total available team time and recorded patient-centred activity reflects the study’s unit of analysis, which captures services delivered to patients rather than total staff workload. As the dataset does not include all team activities, this gap cannot be meaningfully quantified in absolute terms without extending beyond the empirical basis of the study.

The team’s affiliated offices functioned as a coordination hub, extended with a substantial share of contacts conducted via digital communication channels (28%), primarily used for rapid coordination tasks. The phone and text messages also served as a supplement, but not a substitute, to outreaching face-to-face contacts (79% outreach in counts vs. 49% total measured time). The team members noted a higher-than-expected reliance on telephone communication when the quantitative results were presented, and in the focus group, they emphasized that telephone communication was indispensable for maintaining operational efficiency. Although the team demonstrated a high outreach level (79%), meaning most face-to-face contacts occurring outside the office, only 20% of contacts involved travel. Travel durations were generally short, with most lasting 20 min or less. This highlights how outreach-oriented care can be operationalised efficiently through structured planning, potentially reflecting effective logistical organisation in the team.

Services delivered in collaboration with external partners (20%) encompassed psychiatric, somatic, legal, and welfare services, spanning both municipal and specialist levels of care, and also involved employers and relatives. These services represented service areas outside the remit of Norwegian FACT teams [15], and predominantly involved welfare support (NAV) and somatic treatment. Since these services cannot be provided directly by FACT teams, structured collaboration and coordination with external providers is necessary, accounting for more than half of the time allocated to co-produced patient-centered service delivery. Although not directly comparable, this distribution corresponds with findings from a study from the Netherlands [35], that concluded that 30% of a FACT team’s total staff-time, not patient-centered time, was spent on clinical tasks, 30% on coordination and collaboration. In this Dutch study, 13% of services were provided in collaboration with external partners, and 53% of these collaborative contacts involved coordination and interprofessional collaboration.

Our findings show that 37% of total time was allocated to coordination and collaboration activities, in addition to coordination work embedded in FACT board meetings. This aligns with prior research highlighting the largely invisible organisational work required to sustain and mobilise care in complex service systems [26, 27]. By examining these processes from the perspective of team members, our qualitative analysis elucidates the substantial effort required to maintain coherence in patients’ care trajectories, clarifying why coordination and collaboration constitute such a significant investment of time within rural FACT practice. Team members articulated three routine practices that render integrated care feasible under rural resource constraints: collaborative alliance, which entails trust-based partnerships with patients, relatives, and services; information alignment, which involves gathering, refining, and communicating fragmented information so that involved actors can work from a shared and actionable understanding of the patient’s situation; and safety valve thinking, which encompasses anticipating uncertainty and maintaining contingency plans to ensure flexibility and resilience. Under these rural constraints, the CM appears to assume a coordinating role that operates in parallel with established FACT working methods. This local adaptation should not be understood as a shift toward individual case management, but rather as an overarching coordination function embedded within team-based care processes. This interpretation is consistent with findings from previous Norwegian studies [8, 32]. Safety-valve thinking can be understood as a practical articulation of the FACT model’s adaptive capacity, combined with adaptations to rural service delivery. Such adaptations include the introduction of the “back board,” along with proactive strategies for coping with harsh weather conditions and the FACT teams limited office hours in this Norwegian archipelago. *Safety-valve thinking* is not an individual cognitive strategy alone, but an emergent property of collaborative, relational work. *Safety-valve thinking* is contingent upon *information alignment*, a shared and continuously negotiated understanding of the patient’s situation. Such alignment, in turn, relies on the relational and interpersonal conditions embedded in a *collaborative alliance*, including trust, psychological safety, and mutual recognition of expertise. These findings resonate with previous research demonstrating that relational factors are critical for effective collaboration and coordination particularly in contexts characterized by high uncertainty and interdependence [10, 38].

These integrated findings are consistent with challenges widely described in rural mental health services, including fragmented service structures, multi‑level organisation, limited local resources, and poorly integrated digital record systems [8, 11, 24, 30, 45, 46]. While several of these coordination demands are not unique to rural FACT teams, coordination poses distinct challenges in rural settings due to long travel distances, smaller service environments, and reduced access to specialist resources [25]. By explicating the link between collaborative alliance, information alignment, and safety-valve thinking, this study adds nuance to existing accounts of relational coordination. It illustrates how patient safety emerges from situated collaborative practices shaped by both context-specific constraints and broader organisational conditions that influence how care is organised and enacted within the FACT model in a rural network of care.

### Strengths and limitations

We consider the mixed-methods approach to be a central methodological strength. This design enhances interpretability by aligning quantified service patterns in a rural FACT team to exploration of the routines and practices that enable alignment and coherence in the delivery of integrated care. Furthermore, the service mapping used prospective, team-completed registrations across 12 weeks and weighted analyses by time, which may provide a more nuanced picture than contact counts alone.

A key methodological consideration is that recorded time represents patient-centred episode time rather than accumulated staff working hours. Activities involving multiple professionals are therefore expressed as a single unit of patient time. As the data were based on team-reported registrations, variation in reporting practices and potential under- or overestimation of time use cannot be ruled out.

Routine internal team meetings were not included in the quantitative data. While this approach allowed us to focus on patient-related activities, such meetings represent core integrative structures in the FACT model. Their exclusion may therefore lead to an underestimation of coordination work and may influence how the extent and nature of coordination are interpreted. In addition, the qualitative data are based on staff perspectives only, and do not directly capture patient or family experiences. This may influence how relational and collaborative aspects of care are represented.

Furthermore, the absence of systematically recorded data on patient presence represents a limitation of the study. Although indirect estimations were possible, future studies should include patient presence as a predefined variable. In addition, the study does not differentiate between regular care and periods of intensive outreach (ACT-like upscaling), limiting the ability to capture dynamic variations in care intensity.

As the study does not assess model fidelity directly, interpretations of the findings in relation to the normative FACT model should be made with caution. Generalisability is limited by the single-team design and the specific rural context. Nevertheless, context-mechanism links identified here may be transferable to similar sparsely populated settings. Finally, comparison with a urban context would have been a strength.

### Implications for practice and policy

These findings may contribute to addressing the broader challenge of designing service delivery models that can reliably support integrated care within health systems that must adapt to the marked heterogeneity of rural contexts, and perhaps, also of urban contexts. Evaluations of FACT implementation should therefore attend to both model fidelity and context-sensitive adaptations that sustain patient safety and continuity of care.

### Implications for future research

Future studies should (i) validate in-person estimates by capturing patient presence directly, (ii) distinguish between standardized organizational procedures rather than adaptive coordination activities, (iii) compare rural and urban FACT teams to examine context-specific thresholds, (iv) explore how rural adaptations of the FACT model are associated with patient outcomes and the mechanisms underpinning integrated, high-quality care under rural constraints, and (v) systematically capture variations in care intensity, including transitions between regular care and periods of intensive outreach, as well as FACT board utilisation.

## Conclusion

This study demonstrates that a rural Norwegian FACT team provides a comprehensive range of services, with approximately 80% delivered directly by the team and 20% provided in collaboration with external services. Organising care in this context is time-consuming, accounting for 37% of total activity, and is enabled through situated collaborative practices in which the FACT team functions as a coordination hub within a rural network of care. Effective integration was contingent on relational factors, with involved actors relying on a shared frame of understanding to manage uncertainty and complexity under rural conditions. These findings underscore the importance of recognising coordination, relational work, and adaptive local practices as central to the effective enactment of the FACT model in rural settings. Evaluations of FACT implementation should therefore attend to both model fidelity and context-sensitive adaptations that sustain patient safety and continuity of care.

## Supplementary Information

Below is the link to the electronic supplementary material.

Supplementary Material 1

Supplementary Material 2

## Data Availability

Data cannot be shared openly but are available on reasonable request with the corresponding author. The data that support the findings of this study are available from Innlandet Hospital Trust, but restrictions apply to their availability. The data were used under licence for the current study and are therefore not publicly available. The data are, however, available from the corresponding author upon reasonable request and with permission from Innlandet Hospital Trust.

## Abbreviations

ACT: Assertive Community Treatment
CM / CMs: Case Manager / Case Managers
DPS: District Psychiatric Center
FACT: Flexible Assertive Community Treatment
FTE: Full time equivalent
GP / GPs: General Practitioner / General Practitioners
NAV: Norwegian Labour and Welfare Administration
OAT: Opioid Agonist Treatment
SMI: Severe Mental Illness
SMS: Short Message Service (text messaging)
SPSS: IBM SPSS Statistics
SUD / SUDs: Substance Use Disorder / Substance Use Disorders
