# Supplementary material for: Integrated care delivery in a rural Norwegian FACT team: an explanatory sequential mixed-methods study
Source: BMC Health Serv Res. 2026 Jul 18;26:1403. doi: 10.1186/s12913-026-15094-w (PMC13673708; doi:10.1186/s12913-026-15094-w)
Supplement: Supplementary file 1 [file 12913_2026_15094_MOESM1_ESM.pdf]

Supplementary File 1

Semi-structured interview guide: Coordination and collaboration practices of a rural FACT team

Opening

- Brief welcome and study introduction.
- Obtain written informed consent.
- Quick round of introduction of the team members (role in the FACT team).
- Questions based on unexpected findings in the service-mapping data.

1. Use of telephone and SMS

We found extensive use of telephone and SMS in daily work — please reflect upon this.

2. Time spent face-to-face with patients

We found that considerably less time was spent face-to-face with patients — please reflect upon this.

3. Collaboration and coordination practices within the team

We found that over one third of the team's work involved coordination and collaboration — please reflect upon this.

4. Coordination and collaboration with external partners

We found that almost 60% of the team's time with external partners involved coordination and collaboration — please reflect upon this.

5. Final reflections

Please reflect upon anything important that has not yet been discussed.
