# Supplementary material for: Integrated care delivery in a rural Norwegian FACT team: an explanatory sequential mixed-methods study
Source: BMC Health Serv Res. 2026 Jul 18;26:1403. doi: 10.1186/s12913-026-15094-w (PMC13673708; doi:10.1186/s12913-026-15094-w)
Supplement: Supplementary file 2 [file 12913_2026_15094_MOESM2_ESM.pdf]

Date	Location	Patient ID	Other team member(s)	Content (main + additional)		External service provider	Duration (min)	Travel time (min)	Location:
									1 Community based
									2 Team-affiliated office
									3 External service provider office
									4 Phone call
									5 Text message
									6 Videoconference
									**Content**
									1 Initiating contact
									2 Building a relation
									3 Assessment
									4 Treatment planning
									5 Medication review
									6 Crisis intervention
									7 Individual psychotherapy
									8 Supportive therapy
									9 Social skills training
									10 Social and leisure activities
									11 Informal contact with the patient’s network
									12 Family support meeting
									13 Substance use treatment
									14 Vocational and educational support
									15 Housing support
									16 Financial support
									17 Practical support
									18 Accompaniment to medical and other services
									19 Meeting with the patient present
									20 Case discussion and supervision
									21 Care and crisis plan documentation
									22 Coordination with other services
									23 Did not attend/no contact
									24 Other
									**Other participants involved in the contact**
									1 GP
									2 Emergency primary care service
									3 Somatic hospital
									4 Dentist
									5 Municipal mental health and substance use services
									6 Municipal home care services
									7 The Norwegian Labour and Welfare Administration (NAV)
									8 Employers
									9 Outpatient and acute services at District Psychiatric Centre (DPS)
									10 Inpatient unit at DPS or hospital
									11 Substance use outpatient clinic, including opioid agonist treatment (OAT)
									12 Inpatient substance use treatment services
									13 Psychiatrist / psychologist in private practice
									14 Police and correctional service authorities
									15 Voluntary sector
									16 Family and next of kin
									17 Others
